# A Closer Look at the Environmental Impact of Solar and Wind Energy

**DOI:** 10.1002/gch2.202200016

**Published:** 2022-06-22

**Authors:** Jaime Fernández Torres, Fontina Petrakopoulou

**Affiliations:** ^1^ Department of Thermal and Fluid Engineering University Carlos III of Madrid Avda. De la Universidad 30, Leganés Madrid 28913 Spain

**Keywords:** environmental impact, fossil fuels, life cycle assessment, solar energy, wind energy

## Abstract

Moving towards a sustainable society implies constant improvement in the way energy is supplied and consumed, with wider implementation of solar and wind energy facilities in stand‐alone or hybrid configurations. The goal of this work is to evaluate the lifecycle performance (construction and operation‐related impact) of large‐scale solar and wind energy systems and to compare it with conventional coal and natural gas fossil fuel plants under similar conditions. Environmental analyses of energy conversion systems today usually neglect the construction‐related environmental impact of fossil fuel plants, because it is significantly smaller than the impact related to the operation of the plant. However, the construction of large‐scale renewable plants implies the use of rare materials, transport‐related emissions, and other environmentally impactful activities. The plants evaluated here are configured and compared for similar emissions and similar power output. It is found that the life‐cycle environmental impact of the renewable plants could, in some specific cases, exceed that of the fossil fuel plants. Understanding the reasons behind this and the possible limitations of the different technologies can help plan for sustainable energy systems in the future. Finally, solutions to minimize the impact of renewable energy are proposed for more environmentally friendly implementation and future research.

## Graphical abstract

This work presents a comparison of the lifecycle environmental impact of large‐scale renewable and fossil fuel plants under similar conditions. The analysis accounts for the construction, operation, and recycling of the plants. It is seen that renewable power plants may also result in significant impacts that should be minimized to ensure sustainable future applications.

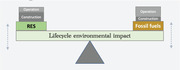

## 1 Introduction

Transportation, electricity, heating, and cooling sectors are driven both by non‐renewable and renewable primary energy sources.^[^ ^1^ ^]^ The main non‐renewable sources are coal, oil, natural gas, and nuclear energy and represent more than 60% of today's global power generation.^[^ ^2^ ^]^ According to the Organization for Economic Co‐operation and Development (OECD), nearly half of the electricity produced in 2020, came from natural gas and coal‐fired power plants.^[^ ^3^ ^]^

Coal has the highest CO_2_ emissions, followed by oil and gas. Although cheap and accessible, the use of coal is being limited because of its significant environmental impact that goes against sustainability and energy targets set for the next decades by countries worldwide.^[^ ^4^, ^5^ ^]^ Natural gas plants emit approximately one‐third of the greenhouse gases (GHG) emitted by conventional coal‐fired plants.^[^ ^6^ ^]^ In 2018, 70% of the emissions in the power sector were released by coal‐fired power plants. This corresponded to approximately 29% of the global CO_2_ emissions. Transportation, largely based on oil, was the second most polluting sector in 2018.^[^ ^2^, ^4^ ^]^ With regard to nuclear power, its low cost and greenhouse gas emissions make it an attractive energy source. However, the radioactive waste and the possibility of a nuclear accident hinder its wider adaptation.^[^ ^5^ ^]^

Among the main types of renewable energy sources (RES), hydropower, wind and solar energy are the most prominent. Hydroelectricity is very efficient and widely deployed, with the highest production share among all renewable technologies.^[^ ^7^ ^]^ The great potential of wind and solar energy systems, however, is expected to increase the importance of these technologies in the future energy mix.^[^ ^8^, ^9^ ^]^ An overview of the state‐of‐the‐art of the main RES types and their basic characteristics can be found in Appendix A.

Today, there is a worldwide push towards the decarbonization of the power and transport sectors. The European Commission has set long‐term energy goals to be climate‐neutral in the next three decades.^[^ ^10^ ^]^ By 2030, the share of renewables in the EU must be 32.5% and the GHG emissions must be decreased by 55%, compared to 1990 levels. Additionally, a 32.5% improvement in energy efficiency must be achieved by that time.^[^ ^11^ ^]^

To set correct goals for a sustainable energy sector, it is necessary to thoroughly study the construction‐ and operation‐related environmental impact of renewable and non‐renewable energy sources (NRES). A well‐defined comparative analysis between the total environmental impact of RES and NRES under similar conditions is still missing. The aim of this study is to critically compare the environmental performance of wind, solar, and fossil fuel plants, including all relevant life cycle stages. On the side of RES, the focus is on manufacturing, construction, and installation. Indirect impacts, like noise or animal disturbance, that intrinsically come with the deployment of renewable energy are not accounted for in this study. With NRES, on the other hand, the focus is mainly on the operation of the plant that is the primary source of emissions.^[^ ^12^, ^13^, ^14^ ^]^ Previous studies present comparative analyses between different energy sources, specifically between wind, coal, nuclear, and hydropower.^[^ ^15^, ^16^, ^17^ ^]^ These works mainly focus on the specific environmental contributions of each energy source, like global warming, acidification, eutrophication, etc. and focus on the most polluting stages over the life cycle of the different power plants. The novelty of this work relies on the comparison of RES and NRES under similar conditions and accounting for all stages of their life cycle. Specifically, the plants evaluated are configured and compared under two different scenarios: the scenario of similar emissions and the scenario of similar power output. The first case involves the study of the power generation of the plants if they had the same overall environmental impact throughout their lifetime. The second case involves the evaluation of the impact of the plants if they generated the same power output throughout their lifetime.

## 2 Life Cycle Assessment of Power Plants Based on Renewable Energy Sources

The evaluation of the environmental impact of solar and wind power plants is based on a wide range of Life Cycle Assessment (LCA) studies. The comparison between RES and NRES power plants with numerical data is realized with studies using the same impact assessment methods and categories of environmental impacts. The chosen studies may focus on different parts of the lifecycle of the power plants. For example, they may present the overall lifecycle of the power plants, that is, from material extraction to decommissioning, or only the impact of certain life cycle stages, such as manufacturing.

### 2.1 Wind Energy

Several LCA studies of wind farms present data on capacity, dimensions of the turbines, type of generators, and location characteristics. The power output of onshore wind farm applications is commonly between 50 and 100 MW,^[^ ^15^, ^17^, ^18^, ^19^, ^20^, ^21^, ^22^, ^23^, ^24^, ^25^ ^]^ while in offshore applications,^[^ ^18^, ^26^, ^27^, ^28^, ^29^ ^]^ the capacities are usually between 300 to 500 MW. Most of the studies included here have been carried out in Europe and some in China and the US.

The contributions of manufacturing, installation, and operation stages of onshore applications to the overall impact are approximately 75%, 15%, and 10%, respectively. The same stages in offshore applications contribute 65%, 25%, and 10%, respectively, to the total environmental impact of a wind facility.^[^ ^17^, ^18^ ^]^ Production processes that involve steel, iron, copper, and composite materials for the tower, nacelle, and rotor, along with the high fuel consumption of the vessels needed for the installation of offshore wind turbines, are responsible for most environmental impact.^[^ ^18^, ^19^ ^]^ Steel and cast iron used for the components of the turbine, strongly contribute to the acidification potential (AP) and the global warming potential (GWP) due to the generation of emissions like sulfur, nitrogen oxides, and carbon dioxide. However, significant impacts are related to the categories of eutrophication potential (EP) and human toxicity potential (HTP) from air and water emissions linked to the use of arsenic, zinc, chromium, and nickel in the production of steel and copper.^[^ ^20^, ^21^ ^]^ Moreover, the emissions during the manufacturing of polymers used in the blades contribute to the ecotoxicity of freshwater significantly.^[^ ^22^ ^]^ The photochemical ozone creation potential (POCP) is particularly affected by emissions of butane, ethane, carbon monoxide, and chlorofluorocarbons during the production of steel, copper, aluminum, and the resins used in the rotor blades.^[^ ^18^, ^23^ ^]^ For onshore wind farms, the tower, the nacelle, and the rotor represent on average 30%, 27%, and 15% of the calculated GWP, respectively,^[^ ^19^ ^]^ whereas the remaining 28% is linked to the concrete foundations, transformers, and the cabling system. In offshore applications, 35% of the GWP stems from the manufacturing of the monopile foundation, followed by the main components of the wind turbine generators (WTG) and the submarine cables.^[^ ^18^, ^26^ ^]^ The GWP of an offshore wind plant reaches, in the best case, the value of 8 g CO_2eq_/kWh.^[^ ^18^ ^]^ In onshore installations, on the other hand, this value could decrease to 5 g CO_2eq_/kWh.^[^ ^20^ ^]^ The main reason for this difference is the greater demand for metals in offshore turbines and their foundations.

Another important factor that influences the impact of wind applications is the generator used. Normally, excited‐synchronous generators have a higher impact, when compared to doubly‐fed induction and permanent magnet‐synchronous technologies, due to their larger weight.^[^ ^24^ ^]^ The first type represents the heaviest option because they are made with large amounts of copper, followed by doubly‐fed induction and permanent magnet‐synchronous generators. The latter is mainly made of iron instead of copper which leads to a weight reduction of the nacelle of approximately 80%. The low weight of this technology implies less material and hence, less demanding production and generated pollutants.^[^ ^22^ ^]^

The recycling of the involved metals leads to an average reduction in all impact categories of 30%.^[^ ^20^, ^21^ ^]^ The higher the recycling rates of the different materials, the best the reported environmental results are.^[^ ^15^, ^17^, ^18^, ^20^, ^21^, ^23^, ^29^ ^]^ High recycling rates usually refer to a recovery of metal components at percentages higher than 90%. Polymer materials used in the blades are recycled at percentages close to 33%, with the remaining 66% sent to landfills.

### 2.2 Solar PV

LCA studies show that, on average, more than 80% of the environmental impact of solar PV is due to the production process of the included modules. Most works^[^ ^30^, ^31^, ^32^, ^33^ ^]^ focus on the manufacturing of different crystalline modules and explain the impacts of this stage, while other studies evaluate the life cycle impacts of rooftop PV systems^[^ ^34^, ^35^, ^36^, ^37^, ^38^, ^39^, ^40^, ^41^, ^42^, ^43^ ^]^ and utility‐scale power plants.^[^ ^44^, ^45^, ^46^, ^47^, ^48^, ^49^, ^50^ ^]^ In general, the results are strongly affected by six parameters: power generation, cell type, efficiency, solar irradiation, lifetime, and electricity mix.^[^ ^30^ ^]^ The manufacturing process of crystalline silicon modules requires a large energy input due to the intensive purification of silicon and wafer processing, especially in the case of monocrystalline silicon (m‐Si) cells.^[^ ^34^ ^]^ Hence, the environmental impact of the process depends strongly on the electricity mix considered in each study. Relevant studies used here are carried out in China, where the share of coal‐based electricity is approximately 60%.^[^ ^16^ ^]^ For example, the work of Y. Fu et al.^[^ ^32^ ^]^ reports that 45% to 80% of the total impact in the production process of a single polycrystalline silicon (p‐Si) module is due to the coal‐based electricity used. The remaining impact is linked to the aluminum frame and the manufacturing of the polymer layers that have high emissions of NO_3_, PO_3_, and SO_2_. Additionally, the emissions of strong solvents during the crystallization process result in a significant acidification impact. For instance, a 1.8 MW PV facility in Italy, with imported modules from China results in a GWP of 88.7 g CO_2eq_/kWh.^[^ ^47^ ^]^ As a comparison, a 5 MW solar plant in France, with European PV modules has a GWP of 37.5 g CO_2eq_/kWh.^[^ ^48^ ^]^ This is also observed in the work of L. Stamford and A. Azapagic,^[^ ^40^ ^]^ where two identical solar roofs of 3 kWp, one manufactured in Germany and another in China, were compared. Specifically, they showed that by changing to a European electricity mix, the GHG emissions of m‐Si and p‐Si modules decreased by 17.6% and 13%, respectively.

The production of the auxiliary systems of a PV plant also results in some environmental impacts. For instance, the work of A. Rashedi and T. Khanam^[^ ^36^ ^]^ shows that in the construction of a 1 kWp p‐Si rooftop PV system, nearly 35% of HTP is due to the manufacturing of the inverter. Moreover, the manufacturing process of the supporting structure is also important because it is commonly made of aluminum or steel. Emissions from the manufacturing of such metals increase the environmental impact categories of GWP, HTP, and APs.^[^ ^48^ ^]^ They also showed that if the modules of their PV system were made of cadmium telluride cells instead of p‐Si, the HTP would be somewhat higher, due to the toxicity of cadmium. Nevertheless, the relatively simple manufacturing and low demand for energy and materials of cadmium telluride, make it the technology with the lowest environmental impact among the different types of solar cells.^[^ ^35^, ^36^ ^]^

The power output of the modules increases with their efficiency. Larger renewable facilities are consequently also related to relatively lower GHG emissions. The work of A. Hamizah Mohd Nordin^[^ ^34^ ^]^ showed that increasing the capacity of a 3kWp m‐Si module to 12 kWp somewhat lowers the GWP from 70 g CO_2eq_/kWh to 65 g CO_2eq_/kWh. Additionally, when the assumed lifetime was changed from 20 to 30 years, the emissions were reduced by 31%, because more renewable power was generated overall. Higher solar irradiation is another impactful factor because it results in more generated power. F. Murphy and K. McDonnell^[^ ^38^ ^]^ calculated that an increase in the irradiation from 963 to 1700 kWh m^−2^ would decrease the GWP of a 3 kWp m‐Si system from 69.6 g CO_2eq_/kWh to 45 g CO_2eq_/kWh.

Finally, recycling can play an important role in the overall environmental analysis of PV plants. From the main materials that make up a solar module, glass, copper, aluminum, silver, and silicon are recycled at an average rate of 85%. Recycling involves remelting and chemical and thermal treatments. Compared to landfill disposal, recycling of crystalline silicon technologies is reported to lower the GWP by 35%.^[^ ^33^ ^]^

### 2.3 Concentrating Solar Power

LCA studies on concentrating solar power (CSP)^[^ ^51^, ^52^, ^53^, ^54^, ^55^, ^56^, ^57^, ^58^, ^59^ ^]^ show that typical solar power tower (SPT) and parabolic trough collector (PTC) plants result in emissions between 20 to 25 g CO_2eq_/kWh. Most environmental impacts of this kind of solar plants are seen to stem equally from the manufacturing and operational stages. The manufacturing phase of a solar‐thermal plant includes the production of the collectors, the heat transfer fluid (HTF), the power block, the necessary pipes, wiring, foundations, etc.^[^ ^51^ ^]^ Materials used include steel, concrete, aluminum, copper, and iron glass used for the mirrors, as well as molten salts and thermal oils.^[^ ^51^ ^]^ In SPT plants, the solar field is responsible for more than 50% of the manufacturing impact, followed by the storage system (tanks and salts) and the receiver, each one with an approximate contribution of 20%. The remaining impact is linked to the power block (i.e., heat exchangers, turbines, pumps, pipes, etc.).^[^ ^52^, ^53^, ^54^ ^]^ In a PTC plant, the distribution is somewhat different. Τhe manufacturing impact of the collectors is found to be around 40% the total impact, that of the storage block around 18%, and that of the production of the thermal oil close to 26%.^[^ ^51^ ^]^

Particularly, the highest manufacturing‐related impacts in solar‐thermal plants are linked to the HTP and GWP. This is again associated with the release of emissions of Cr, Ni, and Zn, as well as NO_x_, SO_x_, and CO_2_ during the production of metals like steel and aluminum.^[^ ^51^ ^]^ C. Mayo et al.^[^ ^55^ ^]^ analyzed the impacts during the manufacturing stage of two different steel materials, used to build the molten salts storage tanks. They showed that the environmental impact of austenitic steel AISI 347H is considerably smaller when compared to the superalloy INCONEL 617. Nearly, 90% of the toxicity released during the production of the latter is due to included metals, like molybdenum or cobalt. AISI 347H is thus seen to be a more suitable material for the metal components of CSP plants. Furthermore, in the works of F.J. Pérez et al.^[^ ^56^ ^]^ and E. Batuecas et al.^[^ ^57^ ^]^ it was found that the HTF Therminol VP‐1 results in a higher environmental impact when compared to molten salts. Approximately, 56 kg of 1.4‐dichlorobenzene equivalents (1.4‐DCB_eq_) and 10 kg of CO_2eq_ were emitted during the production of 1 kg of oil, mainly due to the diphenyl oxide in the eutectic mixture. On the other hand, the production processes of 1 kg of the two commercial types of solar salts, binary and HITEC, were seen to be less polluting than those of thermal oil, releasing 4 and 2 kg of CO_2eq_, respectively.

During the operation stage of a solar‐thermal plant, the sun‐tracking system of the heliostats or collectors, the pumping system of the HTF, and other activities require an electrical input that normally comes from the grid. Hence, the impacts during this phase differ based on the fossil fuel share in the electricity mix assumed.^[^ ^58^ ^]^ Another important aspect linked to environmental impacts is the water needed to keep the mirrors clean and avoid reflectivity losses during maintenance. In addition, the use of a natural gas heater to start the plant and prevent the freezing of the salts further increases the emissions.^[^ ^52^ ^]^ A final factor that has a direct influence on the overall impact of solar‐thermal plants is the solar irradiation. S. Guillén‐Lambea and M. Carvalho^[^ ^59^ ^]^ showed how two similar PTC plants of 100 MW, with 5 h of storage capacity and a lifetime of 30 years located in different countries result in different GWP results. One of the plants was in Northern Cape with an annual mean solar irradiation of 2900 kWh m^−2^, whereas the second one was in California with an annual mean solar irradiation of 2700 kWh m^−2^. The higher irradiation resulted in higher renewable power generation that in turn resulted in lower emissions over the life cycle of the plant.

## 3 Methodology

The environmental impacts accounted for in this work are the GWP expressed in g CO_2eq_/kWh, the HTP in g 1.4–DCB_eq_/kWh, the AP in g SO_2eq_/kWh, the EP in g PO_4eq_/kWh, and the POCP expressed in g C_2_H_4eq_/kWh. The functional unit considered is 1 kWh of electricity generated. In total, 44 papers available on the Scopus database and published between 2016 and 2020 are considered. Additional reports from the International Renewable Energy Agency (IRENA) and the International Energy Agency (IEA) are also used to obtain relevant information. The GWP of NRES, as well as their capacity estimations are based on Refs. [60, 61, 62, 63, 64]. The analysis includes two scenarios: the equivalent power generation and the equivalent environmental impact.

In the case of the equivalent power generation, the design point of the plants is chosen to result in an equal power generation throughout the lifetime of the plants. The annual generation of an NRES plant is calculated by considering continuous, uninterrupted operation (365 days/year and 24 h/day) that is then multiplied by the capacity factor (CF) of the plant. The CF for coal and natural gas plants in this work has been assumed to be equal to 85%. The RES plants are then adjusted to accommodate that annual power generation and the required equivalent capacity is calculated (assuming a CF of 35%, 25%, and 40% for wind power, solar PV, and CSP, respectively).^[^ ^65^, ^66^ ^]^ A conservative 15% reduction in the power output has been accounted for as a CO_2_ capture penalty for plants with carbon capture and storage (CCS).^[^ ^12^ ^]^ The environmental impact of the similar‐sized RES and NRES plants are finally calculated and compared.

In the case of the equivalent environmental impact, the size of the RES plants is increased up to a configuration that would result in a total lifecycle environmental impact equal to that of the fossil fuel plants. This is realized for each one of the five environmental impact categories included in this study. The power generation of the RES and NRES plants is then estimated and compared.

**Table** **1** shows the average specifications of the wind and solar power plants collected from the reports and used as reference plants in this work. **Table** **2** shows the average environmental impact values of the reference plants. The latter includes the impacts of conventional natural gas and coal‐fired power plants based on Refs. [60, 61, 62, 63, 64]. The technologies included have a power output between 500 and 550 MW and are: a natural gas combined cycle (ngcc), a subcritical coal plant (sub coal), a supercritical coal plant (sc coal), and an integrated gasification combined cycle (igcc). There is an additional distinction for plants that include post‐combustion carbon capture with monoethanolamine (MEA),^[^ ^62^ ^]^ implying a GHG reduction of more than 50%.^[^ ^60^ ^]^ Nevertheless, it is seen that other impact categories like acidification, eutrophication, and photochemical ozone creation tend to increase when applying a CCS configuration.^[^ ^60^, ^61^, ^62^ ^]^

**Table 1:** Specifications of the RES plants

Plant Type	Capacity [MW]	Capacity Factor [%]	Lifetime [Years]	Storage [h]	Other	Refs.
Onshore Wind	70	35	20	–	20 WTG of 3.5 MW each. Generator type: DFIG	[10, 12, 32, 33, 34, 35, 36, 37, 38, 39]
PV	196	25	25	–	776 000 p‐Si modules of 245 Wp each	[58, 59, 60, 61, 62, 63, 64]
CSP	150	40	30	7.5	SPT. 7400 heliostats. Molten salts storage.	[65, 66, 67, 68, 69, 70, 71]

**Table 2:** Environmental impacts of reference RES and NRES plants

Plant Type	Capacity [MW]	GWP [gCO_2eq_/kWh]	HTP [g1.4DCB_eq_/kWh]	AP [gSO_2eq_/kWh]	EP [gPO_4eq_/kWh]	POCP [gC_2_H_4eq_/kWh]
Onshore Wind	70	7.34	6.54	0.048	0.038	0.0082
PV	196	68.6	30	0.38	0.13	0.01
CSP	150	24	24.5	0.14	0.01	0.01
NGCC	552	508	88	2.12	0.0054	0.62
NGCC (CCS)	552	207	110	2.6	0.01	0.77
Sub Coal	550	930	110	1.1	0.48	0.81
SC Coal	500	855	150	0.85	0.43	0.2
SC Coal (CCS)	540	410	64	2.36	0.63	1.08
IGCC	497	200	130	0.93	0.58	0.83

## 4 Results

### 4.1 Wind Energy

**Table** **3** presents the results of the first scenario of the study, when the NRES and onshore wind plants generate the same annual power. Overall, it is seen that wind power results in a much lower environmental impact, when compared to coal and natural gas plants. Specifically, the emissions of the wind farms are 36% to 85% lower than the emissions of the coal plants and 32% to 72% lower than the emissions of the NGCC, depending on whether CCS is included in the plant. In general, fossil fuel plants with CCS result in a much lower GWP than the same plants without CO_2_ capture. However, the environmental impacts of the NGCC with CCS, as well as that of the IGCC, are much smaller than those of the other fossil fuel plants, approaching the impacts of the wind alternatives.

**Table 3:** Comparison of the GWP and capacity of wind and NRES plants with the same annual generation of electricity

NRES technology	Annual generation [GWh]	Capacity [MW]		GWP [g CO_2eq_/kWh]	
		NRES	Wind	NRES	Wind
NGCC	4110	552	1341	508	141
NGCC (CCS)	3494	552	1139	207	119
Sub Coal	4095	550	1336	930	140
SC Coal	3723	500	1214	855	127
SC Coal (CCS)	3418	540	1115	410	117
IGCC	3701	497	1207	200	127

*Reference wind farm: Capacity = 70 MW; GWP = 7.34 g CO_2eq_/kWh; Capacity factor: 35%

The influence of the efficiency on the resulting environmental impact is also important to note. The higher efficiency of the SC coal plant leads to a reduction in the GWP of the plant, when compared to that of the Sub coal plant, even when the plants have similar annual generation and capacities.

The resulting capacity of the wind plants is 2–3 times higher than that of the respective NRES plants. For example, to generate the same annual electricity, the installed capacity of wind energy must increase to 1200 MW, when the IGCC and the SC plants can retain capacities of around 500 MW. With the reference wind farm used in this work having a capacity of 70 MW, 4110 GWh would require the equivalent of 19 reference wind farms. This would imply a large surface area requirement in the case of the wind plant.

**Table** **4** shows the results of the second scenario of this work, when the fossil fuel and renewable plants are designed to have the same GWP. It is seen that while the plants result in the same GWP, the wind plants result in a power generation 1.5 to 8 times higher than the fossil fuel alternatives. On the smaller range of that spectrum are the NRES plants with the lowest environmental impacts (NGCC with CSS and IGCC) and on the larger range are sub‐ and SC coal plants. This result depends strongly on the values of CFs assumed. The CFs of the wind plants are relatively low in comparison to the fossil fuel alternatives. However, higher capacities are in turn linked to higher costs and larger surface area requirements. As a quick comparison, an IGCC plant of 497 MW would have the same GWP as a wind farm of 1907 MW, while a sub‐coal‐fired plant of 550 MW would have the same environmental impact of a wind farm of 8869 MW. In addition, the annual electricity generation is significantly higher, as the capacity of the wind plants is substantially higher as well. When compared to the IGCC plant, the annual electricity generation of the wind plant is higher by approximately 60%. Furthermore, the electricity generation of the wind plant is 6 times higher than that of the sub coal plant and 3 times higher than that of the natural gas alternative.

**Table 4:** Comparison of the annual generation and capacity of wind and NRES plants with the same GWP

NRES technology	GWP [g CO_2eq_/kWh]	Capacity [MW]		Annual generation [GWh]	
		NRES	Wind	NRES	Wind
NGCC	508	552	4845	4110	14 854
NGCC (CCS)	207	552	1974	3494	6053
Sub Coal	930	550	8869	4095	27 193
SC Coal	855	500	8154	3723	25 000
SC Coal (CCS)	410	540	3910	3418	11 988
IGCC	200	497	1907	3701	5848

*Reference wind farm: Capacity = 70 MW; GWP = 7.34 g CO_2eq_/kWh; Capacity factor: 35%

Other environmental categories (such as HTP and EP) are not as favorable for wind as GWP. The tables presented in Appendix B show the comparative analysis of the HTP, AP, and POCP impacts. In the case of HTP, the impact of wind power is found to be slightly higher than that of the NRES plants, with the same annual generation. The data show that the environmental performance of wind energy in these categories is better than the performance of some of the NRES plants. In general, an equivalent impact to that of the fossil fuel alternatives, allows a significant increase in renewable electricity production. This increase is found to be between 10% to 65% when the focus is on the AP impact and up to 85% when the POCP is considered.

As seen in **Table** **5**, the same annual power generation results in a slightly higher EP for the wind plants, when compared to most NRES plants. An exception is seen when compared to the SC coal power plant with CCS. In addition, the generation of the same power in the wind plants requires 2–3 times higher capacity when compared to the NRES plants.

**Table 5:** Comparison of the EP and capacity of wind and NRES plants with the same annual generation of electricity

NRES technology	Annual generation [GWh]	Capacity [MW]		EP [g PO_4eq_/kWh]	
		NRES	Wind	NRES	Wind
NGCC	4110	552	1341	0.0054	**0.73**
NGCC (CCS)	3494	552	1139	0.01	**0.62**
Sub Coal	4095	550	1336	0.48	**0.73**
SC Coal	3723	500	1214	0.43	**0.66**
SC Coal (CCS)	3418	540	1115	0.63	0.61
IGCC	3701	497	1207	0.58	**0.66**

*Reference wind farm: Capacity = 70 MW; EP = 0.038 g PO_4eq_/kWh; Capacity factor: 35%

**Table** **6** presents the design of the plants when the EP of the RES and NRES plants is equal. It is seen that a much smaller capacity and annual generation are required in all cases except for the case of the SC plant with CCS. The ratio of the annual generation of the NRES plants over that of the wind plants varies strongly from 1.13 in the case of the IGCC, up to 137 in the case of the natural gas plant. In the worst case, a wind park of 10 MW is seen to have the same EP as one NGCC plant of 552 MW.

**Table 6:** Comparison of the annual generation and capacity of wind and NRES plants with the same EP

NRES technology	EP [g PO_4eq_/kWh]	Capacity [MW]		Annual generation [GWh]	
		NRES	Wind	NRES	Wind
NGCC	0.0054	552	10	4110	**30**
NGCC (CCS)	0.01	552	18	3494	**56**
Sub Coal	0.48	550	884	4095	**2711**
SC Coal	0.43	500	792	3723	**2429**
SC Coal (CCS)	0.63	540	1161	3418	3558
IGCC	0.58	497	1068	3701	**3276**

*Reference wind farm: Capacity = 70 MW; EP = 0.038 g PO_4eq_/kWh; Capacity factor: 35%

### 4.2 Solar PV

**Table** **7** shows the results of the first scenario, where the plants generate the same power output. It is seen that the PV plants result in more than double the capacity of the non‐renewable alternatives. It is also observed that the CO_2_ emissions of the PV plant are lower than those of the sub‐coal (930 g CO_2eq_/kWh) and SC (855 g CO_2eq_/kWh) technologies, but higher than those of the rest of the plants (values shown in bold). When generating the same annual power as the IGCC, the PV plant requires a capacity 3.4 times higher, resulting in approximately 3 times higher emissions (591 vs 200 g CO_2eq_/kWh). This analysis suggests that to perform better than fossil fuel plants with equivalent annual electricity generation, solar PV plants must operate with higher efficiencies and/or be manufactured with a GWP below that of the reference plant used here (68.6 g CO_2eq_/kWh).

**Table 7:** Comparison of the GWP and capacity of PV and NRES plants with the same annual power generation

NRES Technology	Annual Generation [GWh]	Capacity [MW]		GWP [g CO_2eq_/kWh]	
		NRES	PV	NRES	PV
NGCC	4110	552	1877	508	**657**
NGCC (CCS)	3494	552	1595	207	**558**
Sub Coal	4095	550	1870	930	654
SC Coal	3723	500	1700	855	595
SC Coal (CCS)	3418	540	1561	410	**546**
IGCC	3701	497	1690	200	**591**

*Reference PV plant: Capacity = 196 MW; GWP = 68.6 g CO_2eq_/kWh; Capacity factor: 25%

**Table** **8** shows the results of the second scenario when the PV and NRES plants have the same GWP. It is clear that the PV plants result in a higher annual generation, when compared to the coal plants without CCS. In all other cases, the PV plants do not manage to reach the annual power generation of the NRES alternatives, even if their capacities are, in many cases, significantly higher.

**Table 8:** Comparison of the annual generation and capacity of PV and NRES plants with the same GWP

NRES technology	GWP [g CO_2eq_/kWh]	Capacity [MW]		Annual generation [GWh]	
		NRES	PV	NRES	PV
NGCC	508	552	1451	4110	**3179**
NGCC (CCS)	207	552	591	3494	**1295**
Sub Coal	930	550	2657	4095	5819
SC Coal	855	500	2443	3723	5350
SC Coal (CCS)	410	540	1171	3418	**2565**
IGCC	200	497	571	3701	**1251**

*Reference PV plant: Capacity = 196 MW; GWP = 68.6 g CO_2eq_/kWh; Capacity factor: 25%

The results of the analysis of the impacts HTP, AP, and EP are very similar to those of GWP, with a lower difference between PV and NRES capacities, ranging on average between 3% and 60% (Appendix C). In general, these impact values would be smaller if the reference plant had a higher nominal power or if the CF was above 25%.

### 4.3 Concentrating Solar Power

The reference CSP chosen has a thermal storage capacity of 7.5 h, reaching a CF of 40%.

As seen in **Table** **9**, when the CSP and the NRES plants have the same power output (Scenario 1), the capacity of the CSP plants is approximately 2 times higher. The GWP, on the other hand, remains lower for the CSP plants when compared to the NRES alternatives and, in most cases, lower than half that of the NRES plants.

**Table 9:** Comparison of the GWP and capacity of CSP and NRES plants with the same annual power generation

NRES technology	Annual generation [GWh]	Capacity [MW]		GWP [g CO_2eq_/kWh]	
		NRES	CSP	NRES	CSP
NGCC	4110	552	1173	508	188
NGCC (CCS)	3494	552	997	207	160
Sub Coal	4095	550	1169	930	187
SC Coal	3723	500	1063	855	170
SC Coal (CCS)	3418	540	975	410	156
IGCC	3701	497	1056	200	169

*Reference CSP plant: Capacity = 150 MW; GWP = 24 g CO_2eq_/kWh, capacity factor: 40%

**Table** **10** shows the capacity and annual generation of the plants, when the plants result in the same GWP (Scenario 2). It is seen that the annual generation of the CSP is 60% higher than the coal plant with CCS and 80% higher than the sub‐coal plant. In the case of the IGCC and the NGCC with CCS, the annual generation remains slightly lower than that of the CSP plant.

**Table 10:** Comparison of the annual generation and capacity of CSP and NRES plants with the same GWP

NRES technology	GWP [g CO_2eq_/kWh]	Capacity [MW]		Annual generation [GWh]	
		NRES	CSP	NRES	CSP
NGCC	508	552	3175	4110	11 125
NGCC (CCS)	207	552	1294	3494	4533
Sub Coal	930	550	5813	4095	20 367
SC Coal	855	500	5344	3723	18 725
SC Coal (CCS)	410	540	2563	3418	8979
IGCC	200	497	1250	3701	4380

*Reference CSP plant: Capacity = 150 MW; GWP = 24 g CO_2eq_/kWh, capacity factor: 40%

Appendix D presents the results for the remaining environmental impact categories. The worst environmental profile of SPT and PTC plants is found for the HTP. The use of solar salts and thermal oils increases the toxicity potential of CSP technologies considerably and can affect the air and the water if not properly managed. The analysis shows that the HTP of the solar thermal plants ranges between 159 g 1.4‐ DCBeq/kWh and 192 g 1.4‐DCBeq/kWh, emissions 40% higher than those of the fossil fuel plants. This relatively high value is due to the NaNO_3_ composition of the solar salts, which is higher for binary salts than HITEC technology. In the case of a PTC plant, the toxicity is higher because of the use of the Therminol VP‐1 synthetic oil.

Regarding the EP, the CSP plant of 150 MW results in the same value as the NGCC of 552 MW (0.01 g PO_4eq_/kWh), which is the smallest difference found in this category. Hence, although having a higher EP than NGCC for the same annual generation, CSP is the least harmful RES in this category. Compared to the coal plants, the CSP results in an EP 6 to 9 times lower, depending on the technology incorporated. As seen in Appendix D, the CSP presents a better environmental performance than the NRES plants in the impact category of POCP as well.

## 5 Discussion

Overall, wind energy is seen to have the lowest GWP and AP among the renewable plants, followed by concentrating solar thermal and, finally, PV plants. Regarding the HTP, wind energy is again the least polluting technology, while CSP shows the worst results. On the other hand, CSP has the lowest EP, followed in this case by wind and then PV plants. Finally, when analyzing the POCP, PV facilities result in the lowest values, with wind and solar‐thermal plants having a similar impact.

Compared to the fossil fuel plants, CSP and wind plants perform overall better environmentally. On the contrary, PV plants are seen to result in higher values of global warming potential than low‐emission fossil‐fuel plants. Furthermore, renewable plants require significantly higher facilities than fossil fuel plants to result in the same annual power output, due to their relatively lower CFs. Specifically, to achieve the same annual generation as a 500 MW SC coal‐fired power plant, a wind farm of 1214 MW, a PV plant of 1700 MW, or a CSP plant of 1063 MW must be used. The higher relative capacity of the renewable plants can have significant implications on the required surface area for the installation of these plants, that could, consequently, limit their implementation.

Several options can be considered to improve the overall environmental performance of wind and solar energy systems. First, the most effective factor is the recycling rate of the materials used in the manufacturing process. Specifically, wind power plants with recycling rates higher than 90%, recovering metal pieces made of steel, copper, aluminum, and cast iron, used in the nacelle and tower, achieve the best results.^[^ ^18^, ^19^ ^]^ The turbine blades are the most challenging element to recycle as they are commonly made of epoxy or polyester resins. To prevent the blades ending up in landfills, thermoplastic resin blades are now under research as a fully recyclable alternative that significantly reduces the weight and manufacturing cost of the blades.^[^ ^68^, ^69^ ^]^ Particularly, more than 60% of the CFCs emissions are due to the epoxy resin of the blades, increasing the impact of O_3_ depletion.^[^ ^18^ ^]^ The implementation of thermoplastic blades could thus, most probably, largely benefit the environmental performance of wind farms. Regarding the different types of generators, permanent‐magnet synchronous generators (PMSGs) have remarkably better results in all environmental impact categories because of the lower material demand, when compared to doubly‐fed induction generator (DFIG).^[^ ^20^, ^23^ ^]^ The implementation of more PMSG turbines could thus be one of the measures to reduce the environmental impact of the next‐generation wind farms.

In PV plants, the recycling rate should be close to 95% to significantly decrease the environmental impact, especially due to the recovery of silicon wafers and aluminum frames. Moreover, the electricity mix needs to have a high renewable share to reduce the GWP and fossil fuel depletion of the electricity needed in the manufacturing process of PV modules.^[^ ^33^ ^]^ As expected, an increase in the efficiency of solar cells would further reduce the raw materials and consequently lower the overall impacts. Using recycled aluminum and glass to reduce toxic emissions during the production process is another alternative.^[^ ^31^ ^]^ Relying on other technologies, different than crystalline silicon, would also have a strong impact. For instance, cadmium telluride cells are cheaper to produce and have a lower environmental impact than crystalline silicon PV cells, as they require less energy and consume less water.^[^ ^30^ ^]^ Moreover, under humid and warm weather conditions, cadmium telluride cells perform better than crystalline technologies and are also less affected by shadowing.^[^ ^70^ ^]^ Other important parameters that significantly affect the GWP of PV are the lifetime and power output. From the LCA reports reviewed in this work, it is seen that a long lifetime, a larger capacity or high irradiation conditions, result in PV systems with better environmental profiles.^[^ ^34^ ^]^

In CSP plants, salts or synthetic oils are landfilled as toxic waste. Other, more environmentally friendly, HTFs are crucial to improve the environmental performance of CSP plants. Several studies concluded that the HITEC solar salt is the least pollutive among different HTFs, due to its low concentration of NaNO_3_.^[^ ^56^, ^57^ ^]^ In addition, it can be used as storage material in salt tanks. The impact of salt tanks is lower when made of austenitic steel instead of superalloys. Despite the extremely good mechanical and thermal properties of superalloy INCONEL 617, its composition includes toxic metals like molybdenum or cobalt, responsible for more than 90% of the toxicity emitted during the production process. In the solar field, the sun‐tracking system of the heliostats or collectors, the pumping system of the HTF, and other activities require an electrical input that normally comes from the grid. Depending on the fossil fuel share in the electricity mix of the country where the plant is located, the associated impacts differ significantly.^[^ ^58^ ^]^ Finally, if a co‐firing system is included in the plant, the best alternative seems to be biogas derivatives.^[^ ^54^, ^58^, ^71^ ^]^

## 6 Conclusions 

The construction of large‐scale renewable plants involves energy‐demanding processes and significant amounts of rare materials. It was seen that most of the environmental impact of wind and solar plants is linked to manufacturing. In the case of wind energy, the main contributors were the production processes that involve steel, iron, copper, and composite materials for the tower, nacelle, and rotor. For the PV plants, the environmental impact was linked to the production of the included modules and depended strongly on the electricity mix of the manufacturing country. Finally, most of the environmental impact of concentrating solar plants was seen to stem equally from manufacturing and operation (e.g., HTF maintenance, sun‐tracking system).

When compared to fossil fuel alternatives, wind energy was found to have a lower GWP than all fossil‐fuel plants assessed in this study. In the case of PV, on the other hand, that was only true when compared to conventional coal power plants. The GWP of PV was found to be higher than low‐emission technologies like natural gas, integrated gasification, or SC coal with CCS. The GWP of CSP was lower than that of fossil fuel plants. The performance of CSP was better when thermal energy storage was included, leading to more competitive CFs. The natural gas plant with carbon capture and the plant with integrated gasification resulted in a relatively low GWP, close to that of the renewable plants.

It was seen that although renewable plants have near‐zero direct emissions, the environmental impact of large‐scale installations is not negligible, and, in some cases, comparable or even higher than that of low‐carbon fossil fuel plants. Among the factors that can reduce the impacts of wind and solar plants are longer lifetimes, larger power capacities, and higher recycling rates. A plant with a higher capacity and longer lifetime produces larger amounts of energy with lower relative emissions. Hence, the successful transition to renewables should rely on large‐scale plants, with efficient operation and high end‐life recycling rates.

## Appendix A

### State of the Art of RES Technologies

Wind turbine generators use the kinetic energy of wind to move rotor blades and transform the mechanical energy into electricity.^[^ ^18^ ^]^ Wind energy can be installed onshore or offshore. Offshore Wind turbine generators usually imply higher power generation due to more intense gusts of wind, but also higher costs of operation and maintenance. Moreover, they typically require some type of foundation that further increases the mass of the structure and the manufacturing cost.^[^ ^19^ ^]^ The main parts of a wind turbine are the rotor, the nacelle, and the tower. The nacelle includes the low and high‐speed shafts, the generator, the brake, the yaw drive, and the controller. Wind turbines may include a gearbox depending on their type of generator. Gearless configuration (direct drive) does not include any elements between the rotor and the alternator, so they both reach the same rotational speed. The main direct‐drive technologies are excited‐synchronous and permanent‐magnet generator. The most typical technique, however, involves the use of a gearbox to increase the speed of the shaft and produce more power. At utility‐scale, a doubly‐fed induction generator is the most used technology. The deployment of wind energy has gained special attention in China, USA, and some European countries.^[^ ^20^ ^]^ In Europe, Germany leads in onshore wind, whereas the United Kingdom leads in offshore wind applications, with turbines installed all across the North Sea.^[^ ^21^ ^]^

Solar energy systems are divided into PV and solar thermal technologies. Solar PV systems convert sunlight into electricity using the PV effect. Solar panels can be installed on the roof of homes to ensure energy self‐sufficiency, but they can also be used in utility‐scale solar power facilities.^[^ ^22^ ^]^ PV modules are made of solar cells from different materials with particular properties. Typically, solar cells are classified into three main groups. First‐generation cells are made of silicon and are divided into m‐Si or p‐Si. They share almost 95% of the market but p‐Si cells are more dominant due to their easier and cheaper manufacturing.^[^ ^23^ ^]^ The purity of silicon is higher in m‐Si cells, implying a higher efficiency but also a more complex production process. In general, one m‐Si solar module is made of 72 cells, whereas a p‐Si module includes 54 cells, with an average power output of 300 and 200 Wp, respectively.^[^ ^22^, ^23^ ^]^ Second‐generation cells are usually known as thin‐film PV (TFPV). TFPV differs from crystalline cells in the fact that the semi‐conductive material is laminated with very low thickness. This feature makes manufacturing much easier and gives the cell high flexibility. The most developed TFPV cells are cadmium telluride and amorphous silicon. Finally, third‐generation solar cells use organic semiconductors but the level of maturity is really low compared to other types of cells.^[^ ^24^ ^]^ A solar module is normally encapsulated with ethylene vinyl acetate, a polymer material that decreases power losses. A glass layer is produced to protect the module from external environmental elements and then, an aluminum frame is placed to avoid damage.^[^ ^25^ ^]^ The fact that solar modules deliver direct current makes it necessary to include a series of auxiliary elements before the grid connection, such as a charge controller, a DC/AC inverter, a transformer, and a power meter. China, USA, and Japan are the countries with the highest installed capacity of PV.^[^ ^23^ ^]^

Solar‐thermal systems convert sun radiation into thermal energy. One application of such systems is the use of solar collectors for the generation of heat and warm water in buildings. However, solar thermal is more commonly used to generate electricity in central facilities, and specifically in CSP plants. These facilities use special reflectors to concentrate the solar irradiation onto a receiver and heat up a fluid that circulates through it. This fluid can be molten salts or thermal oil that once it reaches the operating temperature, it is used to generate steam and produce electricity in a conventional Rankine cycle.^[^ ^26^ ^]^ The main types of CSP are: PTC, SPT, linear Fresnel reflectors, and parabolic dish collectors.^[^ ^27^ ^]^ Among these, PTC and SPT are the most deployed technologies. PTC is more mature than SPT, but the development potential and efficiency of the latter are higher.^[^ ^28^ ^]^ CSP technologies usually include a thermal energy storage system, commonly based on two molten salts tanks at different temperatures. In existing SPT and PTC plants, common storage capacities are from 10 to 15 h, and 4 to 9 h, respectively.^[^ ^29^ ^]^ World leaders today in installed capacity of CSP are Spain, USA, and China with 48, 15, and 10 operational plants, respectively.^[^ ^30^ ^]^

## Appendix B

Comparison analysis of the HTP, AP, and POCP of wind and NRES power plants.

**Table:** 

NRES Technology	Annual Generation [GWh]	Capacity [MW]		HTP [g 1.4‐DCB_eq_/kWh]		AP [g SO2_eq_/kWh]		POCP [g C_2_H_4eq_/kWh]	
		NRES	Wind	NRES	Wind	NRES	Wind	NRES	Wind
NGCC	4110	552	1341	88	**125**	2.12	0.92	0.62	0.16
NGCC (CCS)	3494	552	1139	110	106	2.6	0.78	0.77	0.13
Sub Coal	4095	550	1336	110	**125**	1.1	0.92	0.81	0.16
SC Coal	3723	500	1214	150	113	0.85	0.83	0.2	0.14
SC Coal (CCS)	3418	540	1115	64	**104**	2.36	0.76	1.08	0.13
IGCC	3701	497	1207	130	113	0.93	0.83	0.83	0.14

**Table:** 

NRES Technology	HTP [g 1.4‐DCB_eq_/kWh]	Capacity [MW]		Annual Generation [GWh]	
		NRES	Wind	NRES	Wind
NGCC	88	552	942	4110	**288**
NGCC (CCS)	110	552	1177	3494	3610
Sub Coal	110	550	1177	4095	**3610**
SC Coal	150	500	1606	3723	4922
SC Coal (CCS)	64	540	685	3418	**2100**
IGCC	130	497	1391	3701	4266

**Table:** 

NRES Technology	AP [g SO2_eq_/kWh]	Capacity [MW]		Annual Generation [GWh]	
		NRES	Wind	NRES	Wind
NGCC	2.12	552	3092	4110	9479
NGCC (CCS)	2.6	552	3792	3494	11 625
Sub Coal	1.1	550	1604	4095	4918
SC Coal	0.85	500	1240	3723	3801
SC Coal (CCS)	2.36	540	3442	3418	10 552
IGCC	0.93	497	1356	3701	4158

## Appendix C

Comparison analysis of the HTP, EP, AP, and POCP of PV and NRES power plants.

**Table:** 

NRES Technology	Annual Generation [GWh]	Capacity [MW]		HTP [g 1.4‐DCB_eq_/kWh]		EP [g PO_4eq_/kWh]		AP [g SO2_eq_/kWh]		POCP [g C_2_H_4eq_/kWh]	
		NRES	PV	NRES	PV	NRES	PV	NRES	PV	NRES	PV
NGCC	4110	552	1877	88	**287**	0.0054	**1.24**	2.12	**3.64**	0.62	0.10
NGCC (CCS)	3494	552	1595	110	**244**	0.01	**1.06**	2.6	**3.09**	0.77	0.08
Sub Coal	4095	550	1870	110	**286**	0.48	**1.24**	1.1	**3.63**	0.81	0.10
SC Coal	3723	500	1700	150	**260**	0.43	**1.13**	0.85	**3.30**	0.2	0.09
SC Coal (CCS)	3418	540	1561	64	**239**	0.63	**1.04**	2.36	**3.03**	1.08	0.08
IGCC	3701	497	1690	130	**259**	0.58	**1.12**	0.93	**3.28**	0.83	0.09

**Table:** 

NRES Technology	HTP [g 1.4‐DCB_eq_/kWh]	Capacity [MW]		Annual Generation [GWh]	
		NRES	PV	NRES	PV
NGCC	88	552	575	4110	**1259**
NGCC (CCS)	110	552	719	3494	**1574**
Sub Coal	110	550	719	4095	**1574**
SC Coal	150	500	980	3723	**2146**
SC Coal (CCS)	64	540	418	3418	**916**
IGCC	130	497	849	3701	**1860**

**Table:** 

NRES Technology	EP [g PO4_eq_/kWh]	Capacity [MW]		Annual Generation [GWh]	
		NRES	PV	NRES	PV
NGCC	0.0054	552	8	4110	**18**
NGCC (CCS)	0.01	552	15	3494	**33**
Sub Coal	0.48	550	724	4095	**1585**
SC Coal	0.43	500	648	3723	**1420**
SC Coal (CCS)	0.63	540	950	3418	**2080**
IGCC	0.58	497	874	3701	**1915**

**Table:** 

NRES Technology	AP [g SO2_eq_/kWh]	Capacity [MW]		Annual Generation [GWh]	
		NRES	PV	NRES	PV
NGCC	2.12	552	1093	4110	**2395**
NGCC (CCS)	2.6	552	1341	3494	**2937**
Sub Coal	1.1	550	567	4095	**1243**
SC Coal	0.85	500	438	3723	**960**
SC Coal (CCS)	2.36	540	1217	3418	**2666**
IGCC	0.93	497	480	3701	**1051**

**Table:** 

NRES Technology	POCP [g C_2_H_4eq_/kWh]	Capacity [MW]		Annual Generation [GWh]	
		NRES	PV	NRES	PV
NGCC	0.62	552	12 152	4110	26 613
NGCC (CCS)	0.77	552	15 092	3494	33 051
Sub Coal	0.81	550	15 876	4095	34 768
SC Coal	0.2	500	3920	3723	8585
SC Coal (CCS)	1.08	540	21 168	3418	46 358
IGCC	0.83	497	16 268	3701	35 627

**Table:** 

NRES Technology	Annual Generation [GWh]	Capacity [MW]		HTP [g 1.4‐DCB_eq_/kWh]		EP [g PO_4eq_/kWh]		AP [g SO2_eq_/kWh]		POCP [g C_2_H_4eq_/kWh]	
		NRES	CSP	NRES	CSP	NRES	CSP	NRES	CSP	NRES	CSP
NGCC	4110	552	1173	88	**192**	0.0054	**0.08**	2.12	1.09	0.62	0.08
NGCC (CCS)	3494	552	997	110	**163**	0.01	**0.07**	2.6	0.93	0.77	0.07
Sub Coal	4095	550	1169	110	**191**	0.48	0.08	1.1	1.09	0.81	0.08
SC Coal	3723	500	1063	150	**174**	0.43	0.07	0.85	**0.99**	0.2	0.07
SC Coal (CCS)	3418	540	975	64	**159**	0.63	0.07	2.36	0.91	1.08	0.07
IGCC	3701	497	1056	130	**173**	0.58	0.07	0.93	**0.99**	0.83	0.07

## Appendix D

Comparison analysis of the HTP, EP, AP, and POCP of CSP and NRES power plants.

**Table:** 

NRES Technology	HTP [g 1.4‐DCB_eq_/kWh]	Capacity [MW]		Annual Generation [GWh]	
		NRES	CSP	NRES	CSP
NGCC	88	552	539	4110	**1888**
NGCC (CCS)	110	552	673	3494	**2360**
Sub Coal	110	550	673	4095	**2360**
SC Coal	150	500	918	3723	**3218**
SC Coal (CCS)	64	540	392	3418	**1373**
IGCC	130	497	796	3701	**2789**

**Table:** 

NRES Technology	EP [g PO4_eq_/kWh]	Capacity [MW]		Annual Generation [GWh]	
		NRES	CSP	NRES	CSP
NGCC	0.0054	552	81	4110	**284**
NGCC (CCS)	0.01	552	150	3494	**526**
Sub Coal	0.48	550	7200	4095	25 229
SC Coal	0.43	500	6450	3723	22 601
SC Coal (CCS)	0.63	540	9450	3418	33 113
IGCC	0.58	497	8700	3701	30 485

**Table:** 

NRES Technology	AP [g SO2_eq_/kWh]	Capacity [MW]		Annual Generation [GWh]	
		NRES	CSP	NRES	CSP
NGCC	2.12	552	2271	4110	7959
NGCC (CCS)	2.6	552	2786	3494	9761
Sub Coal	1.1	550	1179	4095	4130
SC Coal	0.85	500	911	3723	**3191**
SC Coal (CCS)	2.36	540	2529	3418	8860
IGCC	0.93	497	996	3701	**3491**

**Table:** 

NRES Technology	POCP [g C_2_H_4eq_/kWh]	Capacity [MW]		Annual Generation [GWh]	
		NRES	CSP	NRES	CSP
NGCC	0.62	552	9300	4110	32 587
NGCC (CCS)	0.77	552	11 550	3494	40 471
Sub Coal	0.81	550	12 150	4095	42 574
SC Coal	0.2	500	3000	3723	10 512
SC Coal (CCS)	1.08	540	16 200	3418	56 765
IGCC	0.83	497	12 450	3701	43 625

## Conflict of Interest

The authors declare no conflict of interest.

## Author Contributions

J.F.T.: Methodology, Analysis, Writing‐Original Draft, Review & Editing. F.P.: Supervision, Conceptualization, Methodology, Writing‐Original Draft, Review & Editing.

## Data Availability

The data that support the findings of this study are available from the corresponding author upon reasonable request.
